# Accuracy of 23 Equations for Estimating LDL Cholesterol in a Clinical Laboratory Database of 5,051,467 Patients

**DOI:** 10.5334/gh.1214

**Published:** 2023-06-19

**Authors:** Christeen Samuel, Jihwan Park, Aparna Sajja, Erin D. Michos, Roger S. Blumenthal, Steven R. Jones, Seth S. Martin

**Affiliations:** 1 Ciccarone Center for the Prevention of Cardiovascular Disease, Division of Cardiology, Department of Medicine, Johns Hopkins University School of Medicine, Baltimore, MD, USA; 2 Department of Epidemiology, Johns Hopkins Bloomberg School of Public Health, Baltimore, MD, USA; 3 Medstar Georgetown University Hospital-Washington Hospital Center, Division of Cardiology, Washington, DC, USA

**Keywords:** LDL-C, atherosclerotic cardiovascular disease, Friedewald equation, Martin/Hopkins equation, ultracentrifugation

## Abstract

**Background:**

Alternatives to the Friedewald low-density lipoprotein cholesterol (LDL-C) equation have been proposed.

**Objective:**

To compare the accuracy of available LDL-C equations with ultracentrifugation measurement.

**Methods:**

We used the second harvest of the Very Large Database of Lipids (VLDbL), which is a population-representative convenience sample of adult and pediatric patients (N = 5,051,467) with clinical lipid measurements obtained via the vertical auto profile (VAP) ultracentrifugation method between October 1, 2015 and June 30, 2019. We performed a systematic literature review to identify available LDL-C equations and compared their accuracy according to guideline-based classification. We also compared the equations by their median error versus ultracentrifugation. We evaluated LDL-C equations overall and stratified by age, sex, fasting status, and triglyceride levels, as well as in patients with atherosclerotic cardiovascular disease, hypertension, diabetes, kidney disease, inflammation, and thyroid dysfunction.

**Results:**

Analyzing 23 identified LDL-C equations in 5,051,467 patients (mean±SD age, 56±16 years; 53.3% women), the Martin/Hopkins equation most accurately classified LDL-C to the correct category (89.6%), followed by the Sampson (86.3%), Chen (84.4%), Puavilai (84.1%), Delong (83.3%), and Friedewald (83.2%) equations. The other 17 equations were less accurate than Friedewald, with accuracy as low as 35.1%. The median error of equations ranged from –10.8 to 18.7 mg/dL, and was best optimized using the Martin/Hopkins equation (0.3, IQR–1.6 to 2.4 mg/dL). The Martin/Hopkins equation had the highest accuracy after stratifying by age, sex, fasting status, triglyceride levels, and clinical subgroups. In addition, one in five patients who had Friedewald LDL-C <70 mg/dL, and almost half of the patients with Friedewald LDL-C <70 mg/dL and triglyceride levels 150–399 mg/dL, had LDL-C correctly reclassified to >70 mg/dL by the Martin/Hopkins equation.

**Conclusions:**

Most proposed alternatives to the Friedewald equation worsen LDL-C accuracy, and their use could introduce unintended disparities in clinical care. The Martin/Hopkins equation demonstrated the highest LDL-C accuracy overall and across subgroups.

## Introduction

The global burden of cardiovascular disease continued to increase over the past decade and is responsible for an estimated 17 million deaths annually [1]. A major prevention target is low-density lipoprotein cholesterol (LDL-C), a causative factor of atherosclerotic cardiovascular disease (ASCVD) [2]. Reducing LDL-C by 80 mg/dL, or 2 mmol/L, can lower ASCVD risk by 40–50%, and recent clinical guidelines have adopted combination therapy approaches towards lower LDL-C levels [3]. The 2018 American Heart Association/American College of Cardiology (AHA/ACC) guidelines recommend lowering LDL-C levels by ≥50% in patients with ASCVD and intensifying therapy if LDL-C is >70 mg/dL in very-high risk patients [4]. The European Society of Cardiology (ESC) and 2022 ACC non-statin consensus pathway recommend treating LDL-C to <55 mg/dL [5,6]. Clinical laboratories and accrediting institutions have an important role to play in aligning reference values in clinical laboratory reports with the current guidelines to best inform use of lipid lowering therapy to improve outcomes in patients with or at risk for ASCVD.

It is also imperative to have the most accurate means possible of assessing LDL-C to guide evidence-based therapy. Preparative ultracentrifugation, also known as beta quantification (BQ), is the gold standard for measuring LDL-C. However, this method is expensive, laborious, and unsuitable for clinical laboratories performing large volumes of lipid assays daily. An alternative ultracentrifugation-based method is the Vertical Auto Profile (VAP) method, which separates cholesterol into lipoprotein classes using ultracentrifugation and has been validated against BQ with accuracy meeting the requirements of the CDC-NHLBI (Centers for Disease Control-National Heart, Lung, and Blood Institute) Lipid Standardization Program [7,8]. While ultracentrifugation measured LDL-C by BQ or VAP can serve as a reliable reference measure, it is not widely available in clinical practice. Due to lower cost and ease of implementation, computational tools are the method of choice for LDL-C assessment, with the Friedewald equation being the most widely used [9]. The Friedewald equation is generally accurate for the average patient, but underestimates LDL-C at lower levels (especially LDL-C <100 mg/dL), particularly in patients with triglyceride (TG) levels >150 mg/dL, leading to missed prevention opportunities for more aggressive lipid control [10]. In the setting of TG levels >400 mg/dL, Friedewald estimation is increasingly compromised and clinical laboratories often rely on direct chemical-based LDL-C assays [11]. However, these assays add cost, and they lack standardization, accuracy, and traceability [12]. Among patients with dyslipidemia, where accuracy is of the greatest importance in guiding treatment, direct chemical-based LDL-C assays have especially unreliable accuracy [13]. Accuracy of the Friedewald equation is further impaired by non-fasting, while guidelines for clinical practice have increasingly endorsed added flexibility for testing in the non-fasting state [14].

With the current therapeutic armamentarium and the shortcomings of Friedewald estimation becoming more widely known, groups across the world have revisited their country’s LDL-C estimation approach [11,12,14,15]. A steady stream of new equations has entered the literature, attempting to replace the Friedewald equation, with these equations commonly using direct chemical-based assays as a reference LDL-C as opposed to BQ or VAP [11]. Many of these equations have not been externally validated, especially in a large, representative clinical population using ultracentrifugation as the reference. Several prior studies have evaluated a smaller subset of the new equations in various populations, most commonly comparing with direct chemical-based assays [16,17,18]. Due to the aforementioned concerns with these assays, conclusions from these prior studies are limited. Additionally, clinical adoption of new LDL-C equations without sufficient external validation could introduce unintended disparities in clinical care based on geography or access to clinical resources.

Therefore, we sought to identify LDL-C equations reported since 1972 and to compare the accuracy of these equations to ultracentrifugation-measured LDL-C by VAP in a large-scale analysis.

## Methods

### Search for Relevant Studies

The literature published through September 2021 was reviewed by searching PubMed and Web of Science. The following search strategy was developed with the input of a Johns Hopkins Welch Medical Library informationist: (measur*[ti] OR calculat*[ti] OR equation OR formula) AND (low density lipoprotein cholesterol OR LDL-C) in both databases. The initial search yielded 15,477 articles (Supplemental Figure 1), published from 1972 through 2021. Articles were evaluated for their report of new LDL-C equations. The reference lists of the final 23 articles were searched, and no additional LDL-C equations were identified.

### Study Population

This study used data from the second harvest of the Very Large Database of Lipids (VLDbL), registered at http://www.clinicaltrials.gov (NCT01698489). The design of the VLDbL has been described in detail previously [19]. Patient samples were acquired by the VAP Diagnostics Laboratory from October 1, 2015 through June 30, 2019 in the United States. The study included both sexes, as well as children (aged <11 years), adolescents (aged 11 to <18 years), and adults (aged ≥18 years). Patient samples largely originated from primary care clinics (85%), with the remainder from inpatient settings and specialty centers. The lipid distributions in the VLDbL have been shown to reflect those in the population-representative National Health and Nutrition Examination Survey [19]. We excluded samples with missing total cholesterol (TC), high density low-density cholesterol (HDL-C), TG, and LDL-C. We included all samples regardless of their fasting status. The Johns Hopkins Institutional Review Board declared our study exempt.

### Lipid Measurements

The VLDbL includes direct measurements of TC, LDL-C, very low-density lipoprotein cholesterol (VLDL-C), HDL-C, and other lipoprotein parameters. Measurements were performed by VAP to separate lipoprotein classes by density into all five classes (HDL, LDL-Real [LDL-R; the LDL without Lp(a) and IDL], VLDL, IDL, and Lp(a)) and then measure the cholesterol component via enzymatic analysis and spectrophotometric absorbance. The accuracy of VAP was validated by annual yearly random split-sample comparisons with preparative ultracentrifugation or BQ at the Washington University in St. Louis Core Laboratory for Clinical Studies (r = 0.986, r = 0.969, respectively; bias 0.6%, 1.7%, respectively). Correlation coefficients from an analysis using 40 split serum specimens comparing VAP to BQ were: 0.99 for total cholesterol, 0.99 for HDL-C, 0.98 for LDL-C, and 0.98 for VLDL-C [20]. TG levels were directly measured with the Architect C-8000 system (Abbott Laboratories, IL), which were compared with samples from the University of Alabama School of Medicine for quality assessment.

### Equations for Estimating LDL-C

A total of 23 equations for estimating LDL-C are summarized in Supplemental Table 1. One class of LDL-C equations uses a fixed TG:VLDL-C ratio to estimate VLDL-C and extract estimated VLDL-C from non-HDL-C to estimate the LDL-C levels (Friedewald et al. [9], Puavilai et al. [21], Vujovic et al. [22], DeLong et al. [23], Ephraim et al. [24], Ghasemi et al. [25], Bauer et al. [26]). Another class of the equations uses TC, HDL-C, and TG levels as coefficients of linear models to estimate LDL-C (Hattori et al. [27], Anandaraja et al. [28], Chen et al. [29], Cordova et al. [30], Teerakanchana et al. [31], Ahmadi et al. [32], Rao et al. [33], Dansethakul et al. [34], Rasouli et al. [35], Lee & Hu [36], Choi et al. [37], Orejon et al. [38], and Molavi et al. [39]). Other equations use an adjustable factor for TG:VLDL-C ratio based on a lookup table (Martin et al. [40]) or interaction/quadratic terms (Sampson et al. [41], Saiedullah et al. [42]) to account for non-linearity. Furthermore, we used the extended Martin/Hopkins equation [43] for LDL-C estimation at high TG levels (400–799 mg/dL).

### Statistical Analysis

We assessed the accuracy of the 23 equations for estimating LDL-C using VAP ultracentrifugation as the reference. Firstly, we compared the concordance in classification according to clinical guideline-based categories (<40, 40–54, 55–69, 70–99, 100–129, 130–159, 160–189, and ≥190 mg/dL) in patients with TG levels up to 399 mg/dL. Concordance was defined as the proportion of ultracentrifugation-measured LDL-C levels falling in the same category as estimated LDL-C. Secondly, we compared the magnitude of error of each equation compared to ultracentrifugation using median differences.

**Figure 1:**
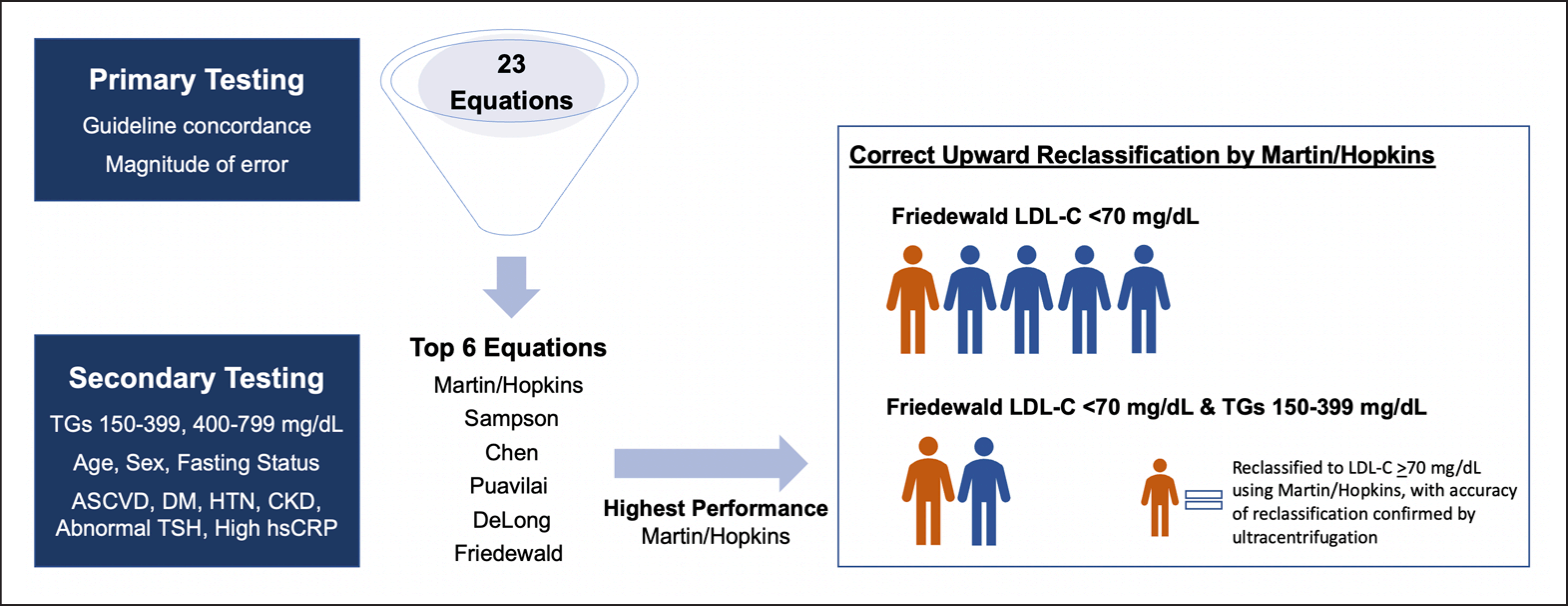
**Diagram of Primary and Secondary Testing to Select the Highest Performing Equation.** The figure shows the sequence of analysis, first comparing all 23 equations against ultracentrifugation for concordance in guideline-based LDL-C classification and overall magnitude of error (mg/dL units). In secondary testing, we compared Friedewald and the five equations that performed better than Friedewald in primary testing. These secondary tests evaluated performance at different levels of elevated triglycerides, by age (<18, 18–59, >60 years), sex, and fasting status strata, and across clinical subgroups (ASCVD, DM, HTN, CKD, abnormal TSH, high hsCRP). Finally, we assessed the top performing equation (Martin/Hopkins) for its impact in classification across an important clinical cutpoint in high risk patients (LDL-C 70 mg/dL) if a laboratory were to switch from the Friedewald equation. Patients highlighted in orange are ones with Friedewald LDL-C<70 mg/dL who have correct (confirmed by ultracentrifugation) upward reclassification to LDL-C >70 mg/dL by Martin-Hopkins. Patients highlighted in blue remain classified as LDL-C <70 mg/dL by both equations. Abbreviations: TGs = triglycerides; ASCVD = atherosclerotic cardiovascular disease; DM = diabetes mellitus; HTN = hypertension; CKD = chronic kidney disease; TSH = thyroid stimulating hormone; hsCRP = high-sensitivity C-reactive protein; LDL-C = low-density lipoprotein cholesterol.

We separated the equations based on whether they performed better than Friedewald (Figure 1). We then conducted secondary analyses for the top equations as well as the Friedewald equation with ultracentrifugation as reference. We first compared the concordance of each equation in patients with TG levels of 150–399 mg/dL and 400–799 mg/dL. We further evaluated LDL-C equations stratified by age (<18, 18–59, ≥60), sex, and fasting status. We also performed analyses stratified by ASCVD (ICD-9 code 410.XX, 414.0X, 411.1, 433.XX, 434.XX, 435.9, 440.2X, 443.9) and hypertension (ICD-9 code 401.XX), and in patients with available laboratory data, we performed analyses stratified by kidney disease (eGFR <60 mL/min/1.73 m^2^), diabetes (ICD-9 250.XX, A1c >6.5%, or fasting glucose ≥126 mg/dL), inflammation (high-sensitivity C-reactive protein ≥ 2 mg/L), and thyroid dysfunction (TSH <0.5 or >4.5 uIU/mL). Finally, we evaluated the percentage of patients with Friedewald LDL-C <70 mg/dL correctly reclassified (confirmed by ultracentrifugation) to LDL-C >70 mg/dL using the other top performing equations.

All statistical analyses were performed in Stata (StataCorp), version 15.1. Graphs were plotted using R (R Core Team) version 4.0.3.

## Results

### Characteristics of the Study Population

A total of 5,051,467 patients were analyzed with 4,939,528 patients included in the primary analyses of patients with TG levels up to 399 mg/dL. The mean (SD) age was 56±16 years and 53.3% of patients were women. The median directly measured LDL-C was 114 (IQR: 90–141) mg/dL and the median TG level was 116 (IQR: 82–169) mg/dL. Considering fasting status, 11.9% of patients were non-fasting, 19.4% were fasting, and we did not have data on fasting status for 68.7% of patients (Table 1). For patient subgroups, 32,223 had ASCVD, 274,286 had hypertension, 361,079 had kidney disease, 212,671 had diabetes, 417,561 had inflammation, 39,515 had TSH <0.5 uIU/mL and 27,350 had TSH ≥4.5 uIU/mL.

**Table 1:** Demographic characteristics.


CHARACTERISTICS	STUDY POPULATION (N = 4,939,528)

Age, mean (SD), y	56 (16)

Age category, no. (%), y	

<18	59,838 (1.2)

18–59	2,879,747 (58.3)

≥60	1,966,468 (39.8)

Not reported	33,475 (0.7)

Sex, no. (%)	

Women	2,635,486 (53.7)

Men	2,269,406 (46.3)

Fasting status, no. (%)	

Non-fasting	586,256 (11.9)

Fasting	958,989 (19.4)

Not reported	3,394,283 (68.7)

Lipid values, median (IQR)	

TC, mg/dL	193 (164–225)

TG, mg/dL	114 (81–164)

HDL-C, mg/dL	51 (42–63)

LDL-C, mg/dL (ultracentrifugation)	114 (90–141)

Non-HDL-C, mg/dL	138 (112–168)

VLDL-C, mg/dL	22 (17–29)

Lp(a)-C, mg/dL	6 (4–10)

TC:VLDL-C ratio	8.4 (6.5–10.9)

TG:VLDL-C ratio	5.0 (4.4–5.9)

TG:TC ratio	0.6 (0.4–0.9)


IQR = interquartile range; TC = total cholesterol; HDL-C = high-density lipoprotein cholesterol; LDL-C = low-density lipoprotein cholesterol; VLDL-C = very low-density lipoprotein cholesterol; Lp(a)-C = lipoprotein(a) cholesterol.

### Concordance According to Clinical LDL-C Classification Guidelines

Among the LDL-C estimation methods, the Martin/Hopkins algorithm most accurately classified LDL-C to the correct clinical category (89.6%), followed by the Sampson (86.3%), Chen (84.4%), Puavilai (84.1%), Delong (83.3%), and Friedewald (83.2%) equations (Table 2). The other 17 equations were less accurate than Friedewald, with accuracy as low as 35.1%.

**Table 2:** Percentage of patients correctly classified to LDL-C category.


EQUATION	LDL-C CATEGORY, MG/DL								

	<40	40–54	55–69	70–99	100–129	130–159	160–189	≥190	OVERALL

Martin/Hopkins	78.6	81.2	84.2	91.7	90.8	89.2	86.6	91.1	89.6

Sampson	66.0	71.8	77.8	90.5	89.0	85.4	80.5	84.4	86.3

Chen	85.9	81.1	80.2	86.0	84.3	83.2	81.7	95.7	84.4

Puavilai	62.5	69.7	75.0	89.3	87.2	83.1	77.0	79.2	84.1

DeLong	66.8	72.3	76.2	89.6	86.4	81.4	74.4	76.8	83.3

Friedewald	42.1	52.5	62.6	83.8	87.0	87.7	86.1	90.0	83.2

Molavi	44.6	54.2	63.0	82.8	85.6	87.2	87.6	95.2	82.9

Saiedullah	72.2	65.8	66.0	81.1	83.6	85.8	86.6	95.4	82.1

Vujovic	75.7	76.3	77.0	89.2	83.6	76.6	68.0	71.2	80.3

Teerakanchana	83.5	70.8	67.0	83.8	78.4	76.6	75.7	83.8	78.5

Orejon	71.5	67.8	68.0	85.3	80.4	76.1	71.1	76.4	78.5

Dansethakul	52.1	58.1	62.1	82.7	77.9	72.9	67.1	73.3	75.2

Bauer	87.2	77.6	73.6	86.5	77.5	68.3	57.8	63.2	73.8

Rao	42.3	54.2	61.8	82.7	76.4	67.3	56.4	62.7	71.0

Ghasemi	21.6	28.9	38.8	68.0	75.5	80.5	83.3	97.8	69.1

Ephraim	93.0	74.3	67.0	83.0	71.7	61.1	49.8	57.3	67.5

Hattori	33.9	38.1	43.5	67.7	70.9	73.1	73.9	99.1	67.0

Lee and Hu	81.4	63.4	57.8	74.0	66.0	63.4	61.5	81.9	66.9

Rasouli	74.9	65.4	62.3	68.1	59.8	48.6	29.0	99.5	60.3

Cordova	86.8	64.5	54.6	62.6	56.6	50.1	41.1	96.7	57.5

Anandaraja	29.9	36.0	43.3	67.6	59.4	52.1	45.3	56.5	55.9

Choi	88.3	56.1	44.4	73.0	57.9	46.7	36.2	49.2	53.4

Ahmadi	27.5	26.0	31.8	58.7	49.1	34.8	18.8	16.5	35.1


### Magnitude of Patient-Level Error

The median error of equations ranged from –10.8 to 18.7 mg/dL, and was best optimized using the Martin/Hopkins equation (0.3, IQR–1.6 to 2.4 mg/dL) (Table 3). In comparison, other equations that demonstrated higher overall accuracy compared to Friedewald had median differences of 1.7 mg/dL (Sampson), 3.3 mg/dL (Puavilai), 4.1 mg/dL (Delong), and –2.9 mg/dL (Chen). In patients with TG levels <400 mg/dL, 68.6% of patients had less than 5 mg/dL error using Friedewald compared to 70.8% and 82.8% using Sampson and Martin/Hopkins, respectively.

**Table 3:** Error between estimated LDL-C and VAP ultracentrifugation LDL-C.


EQUATION	ERROR, MEDIAN (IQR), MG/DL	RELATIVE ERROR, MEDIAN (IQR), %

Friedewald	–0.2 (–4.4 to 2.6)	–0.2 (–4.0 to 2.2)

Martin/Hopkins	0.3 (–1.6 to 2.4)	0.2 (–1.4 to 2.1)

Sampson	1.7 (–1.3 to 4.1)	1.6 (–1.2 to 3.5)

Puavilai	3.3 (0.5 to 5.5)	2.9 (0.4 to 4.9)

Vujovic	5.5 (3.2 to 7.7)	4.8 (2.8 to 6.9)

Hattori	–7.6 (–11.8 to –4.6)	–6.4 (–10.0 to –4.1)

Anandaraja	5.9 (–4.3 to 17.5)	5.1 (–3.6 to 16.1)

Chen	–2.9 (–5.6 to –0.2)	–2.6 (–4.4 to –0.3)

Cordova	–10.8 (–17.2 to –4.5)	–9.8 (–13.4 to –4.7)

Teerakanchana	5.2 (1.7 to 8.9)	4.6 (1.3 to 8.7)

Ahmadi	18.7 (–2.6 to 50.6)	16.4 (–2.3 to 44.6)

DeLong	4.1 (1.4 to 6.2)	3.5 (1.2 to 5.5)

Rao	7.7 (2.3 to 11.6)	6.8 (2.1 to 9.9)

Ephraim	9.4 (7.3 to 12.2)	8.4 (6.4 to 11.1)

Saiedullah	–3.9 (–6.0 to –1.5)	–3.4 (–5.3 to –1.3)

Dansethakul	6.9 (2.7 to 9.7)	5.8 (2.2 to 8.8)

Rasouli	–10.1 (–16.6 to –4.0)	–8.8 (–12.3 to –4.3)

Ghasemi	–5.8 (–12.0 to –1.8)	–5.1 (–11.0 to –1.6)

Lee and Hu	3.8 (–3.8 to 12.0)	3.4 (–3.0 to 12.0)

Orejon	6.2 (3.4 to 8.3)	5.3 (2.7 to 8.0)

Bauer	7.6 (5.6 to 10.1)	6.8 (4.9 to 9.1)

Molavi	–1.1 (–5.2 to 1.7)	–0.9 (–4.5 to 1.5)

Choi	14.5 (11.8 to 17.2)	12.6 (9.8 to 16.0)


### Accuracy of Top Performing Equations at High Triglyceride Levels

In patients with TG levels 150–399 mg/dL, the Martin/Hopkins continued to be the best performing equation with accuracy of 83.5%, followed by Chen (81.3%), DeLong (80.0%), Puavilai (79.4%), Sampson (79.3%), and Friedewald (67.8%) (Supplemental Table 2). In patients with TG levels 400–799 mg/dL (n = 111,939), the extended Martin/Hopkins [43] had the highest accuracy (60.3%), followed by Martin/Hopkins (59.2%) and Chen (57.7%) (Supplemental Table 3). Some equations that demonstrated greater accuracy in patients with TG levels <400 mg/dL performed poorly in patients with hypertriglyceridemia. DeLong had a concordance of 42.2% and Sampson had a concordance of 37.3%.

### Accuracy of Top Performing Equations in Patient Subgroups

The Martin/Hopkins equation was consistently the highest performing equation after stratifying by age, sex, and fasting status. The same pattern was observed when stratifying by ASCVD, hypertension, kidney disease, diabetes, inflammation, and thyroid dysfunction (Supplemental Table 4).

### Percentage of Patients with Friedewald LDL-C <70 mg/dL Reclassified to LDL-C >70 mg/dL Using the Top Performing Equations

**Figure 2:**
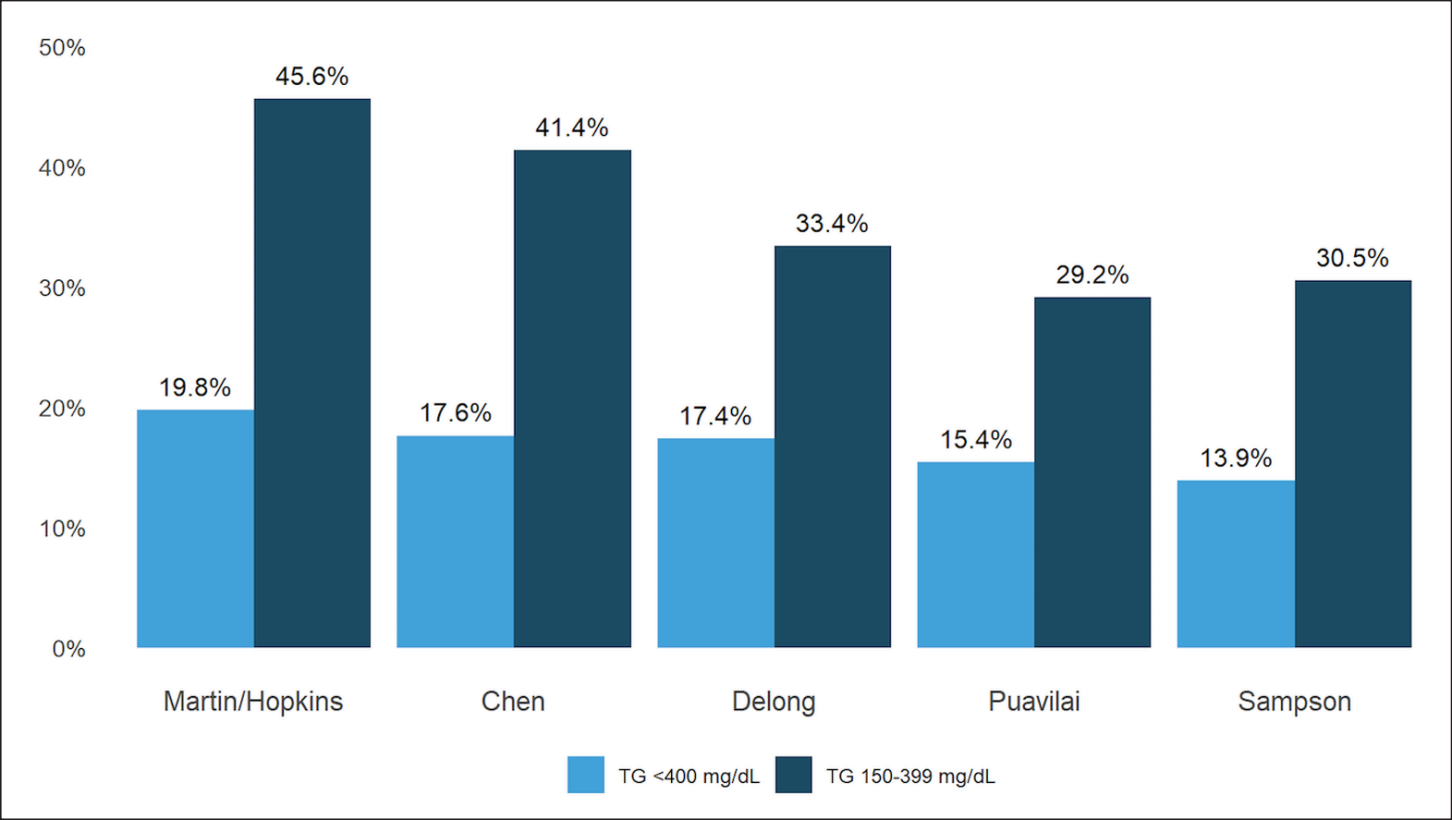
**Upward Reclassification of Patients with Friedewald LDL-C < 70 mg/dL When Using an Alternative LDL-C Equation.** The figure displays the percentage of patients with Friedewald LDL-C < 70 mg/dL who are reclassified to LDL-C > 70 mg/dL by top performing equations and confirmed to have a correct reclassification by ultracentrifugation. Reclassification for patients with TG levels of <400 mg/dL (n = 539,575) is highlighted in blue and for patients with TG levels of 150–399 mg/dL (n = 183,455) is highlighted in dark blue. Abbreviations: LDL-C = low-density lipoprotein cholesterol; TG = triglyceride.

In patients with Friedewald LDL-C <70 mg/dL and TG levels <400 mg/dL, 19.8% were correctly reclassified to LDL-C >70 mg/dL using Martin/Hopkins, 17.6% using Chen, 17.4% using DeLong, 15.4% using Puavilai, and 13.9% using Sampson (Figure 2). In patients with Friedewald LDL-C <70 mg/dL and TG levels 150–399 mg/dL, a considerably greater percentage of patients were correctly reclassified to LDL-C >70 mg/dL, with the Martin/Hopkins having the highest reclassification percentage of 45.6%.

## Discussion

This study compared the performance of 23 LDL-C equations and generated several important findings: 1) most proposed alternatives to the Friedewald equation worsened accuracy of LDL-C; 2) the Martin/Hopkins most accurately classified LDL-C to the correct guideline-based LDL-C category followed by Sampson, Chen, Puavilai, Delong, and Friedewald, respectively; 3) across age, sex, TG levels, and patient subgroups, the Martin/Hopkins continued to have the highest accuracy; 4) the magnitude of error between calculated LDL-C and directly measured LDL-C was the smallest using the Martin/Hopkins compared with the other 22 equations; and 5) approximately one in five patients with Friedewald LDL-C <70 mg/dL and almost half of the patients with Friedewald LDL-C <70 mg/dL and TG levels 150–399 mg/dL were correctly reclassified to LDL-C >70 mg/dL using the Martin/Hopkins equation.

### Approaches to LDL-C Determination

LDL-C is defined as TC – HDL-C – VLDL-C according to the landmark Friedewald equation [9] and the Centers for Disease and Prevention (CDC) definition of LDL-C. The two variables that are directly measured are TC and HDL-C, whereas VLDL-C is estimated. The Friedewald equation estimates VLDL-C using a fixed factor, thus assuming a consistent relationship between VLDL-C and TG levels. Some equations retain the basic backbone of Friedewald, which translates into a permutation of equations that substitute the factor 5 in mg/dL units with other predetermined ratios. For example, DeLong et al. proposed dividing TG levels by 6 [23] while Chen et al. in 2010 suggested that LDL-C = TC × 0.9 – HDL- C × 0.9 – TG × 0.1 [29]. These approaches do not account for heterogeneity in TG:VLDL-C ratios, which limits the capacity for substantial accuracy enhancement.

Other equations have deviated from the structure of the Friedewald equation.De Cordova et al. proposed that LDL-C = 3/4 (TC – HDL-C), which eliminated TG levels as an input variable and VLDL-C estimation [29,30]. Some equations, such as Anandaraja and Lee & Hu, do not include HDL-C as a component in their equations [28,36]. These equations seek to estimate LDL-C directly, which is unlike the focus of Friedewald on estimating VLDL-C to determine LDL-C. Friedewald LDL-C has guided key clinical trials that have shaped treatment recommendations for ASCVD patients [44]. Therefore, direct LDL-C estimation, which drifts away from the standard set by Friedewald, is inconsistent with our clinical standard for LDL-C.

In our analysis, all the equations that estimated LDL-C directly, except Sampson, performed poorly compared to Friedewald. Sampson used a bivariate equation, which included all the input variables used in Friedewald, unlike other equations that dropped key components of the original formula. Although Sampson demonstrated better accuracy than Friedewald, its deviation from the structure of Friedewald still poses the risk of a fundamental discrepancy—the definition of LDL-C used in treatment guidelines would not align with the one used in LDL-C calculation under Sampson. Furthermore, a subset of the equations assumed a non-zero baseline value by adding a y-intercept in their calculation of LDL-C from TC [28,31,32,34,38,42]. Such an approach implies that patients present with a standard pre-existing level of cholesterol, an assumption that may not have merit.

Of the 23 equations, the Martin/Hopkins demonstrated the highest accuracy across strata by age, sex, fasting status, and triglyceride levels, as well as in patients with atherosclerotic cardiovascular disease, hypertension, diabetes, kidney disease, inflammation, and thyroid dysfunction. The Martin/Hopkins equation has been validated extensively on a global scale [11,16,45,46,47]. Martin/Hopkins is the only formula to preserve the structure of Friedewald while leveraging an adjustable factor that improves VLDL-C estimation, catalyzing a more personalized risk assessment [40]. It thus maintains the same LDL-C definition that guided treatment trials, which offers internal consistency in treatment recommendations and calculation methods. The Martin-Hopkins equation was further validated across a wide spectrum of LDL-C levels, including LDL-C <70 mg/dL, using BQ in large-scale studies [10,48]. Given the development of the Martin-Hopkins equation based on VAP, these studies provide further validation of the VAP method.

The adjustable factor in the Martin/Hopkins, which can range from 3.1 to 9.5 in patients with TG levels <400 mg/dL, was derived from an analysis of TG-to-VLDL-C ratios in more than 1.3 million people, as opposed to the Friedewald equation which was derived in a sample of 448 patients [9,40]. Other independent, multi-national groups have reported that the Martin/Hopkins equation was more accurate than Friedewald in racially diverse populations [11,45,49] as well as for LDL-C values <70 mg/dL [11,47], and in patients taking proprotein convertase subtilisin/kexin (PCSK9) inhibitors [10], patients with diabetes [50], and patients with familial combined hyperlipidemia [51].

Various large laboratories have adopted the Martin/Hopkins equation, and the AHA/ACC/Multi-society Cholesterol Guideline provided a Class IIa recommendation for using the equation in patients with LDL-C <70 mg/dL [12]. The Martin/Hopkins equation has been supported by the National Lipid Association (NLA) [52], a consensus recommendation in Brazil [53], a joint consensus panel of the European Atherosclerosis Society (EAS), and the European Federation of Clinical Chemistry and Laboratory Medicine (EFLM) [54]. It was also supported by the World Heart Federation Cholesterol Roadmap in 2022 [55]. It can be installed on lab information systems in a straightforward manner using line-by-line code for automatic calculation of LDL-C. There are no intellectual property restrictions. Laboratories can contact JHTT-Communications@jh.edu for assistance in implementation.

### LDL-C Risk Reclassification at Low LDL-C Levels

We found that if a clinical laboratory were to switch from Friedewald to Martin-Hopkins, almost half of the patients with Friedewald LDL-C <70 mg/dL and TG levels 150–399 mg/dL would be correctly reclassified to LDL-C >70 mg/dL using Martin/Hopkins. Doing so would facilitate opportunities to further optimize LDL-C and ASCVD risk. Our results are consistent with prior studies reporting LDL-C underestimation using Friedewald estimation [10,47,56].

### LDL-C Estimation in Hypertriglyceridemia

In patients with TG levels up to 799 mg/dL, the extended Martin/Hopkins performance was the most accurate. However, at such elevated TG levels, the clinical priority is to immediately reduce TG levels to prevent pancreatitis. Our results align with a previous study demonstrating that while the extended Martin/Hopkins is the most accurate of our LDL-C estimation tools at high TG levels, caution with LDL-C estimation in this elevated TG range should still be taken [43].

### Limitations

This study has some limitations. Data on race and ethnicity or other clinical factors such as body mass index were not available for analysis due to using a clinical laboratory dataset. Our data also comes entirely from a US population. However, multiple national and international studies, including the ELSA Brazil study and a clinical trial conducted in 49 countries, reported results consistent with ours, which serves as an external validation of our findings [10,47,57]. Furthermore, we did not have patient treatment data. Accuracy of LDL-C estimation is important, nonetheless, both pre-treatment and on-treatment. A PCSK9 inhibitor trial and a CETP inhibitor trial showed better accuracy with Martin/Hopkins compared to Friedewald estimation [10,47].

Some studies suggested that LDL-C levels may vary by patients’ clinical characteristics [58,59,60]. We were able to use common biomarkers, such as HbA1c, hsCRP, TSH, and eGFR, as well as ICD codes to assess equation accuracy in subgroup analyses. We found consistent results across these groups. Finally, we used the VAP method as opposed to BQ to directly measure LDL-C. As noted above, studies have demonstrated excellent agreement between the two methods, which are both based on ultracentrifugation. Limited, inconclusive data has raised concern regarding the VAP method and its tendency to underestimate VLDL-C in samples with high TG due to adherence of TG-rich lipoproteins to the walls of test tubes [41,61,62]. However, this concern applies to all forms of ultracentrifugation and is only relevant at TG levels beyond the range in our analysis.

## Conclusion

The influx of LDL-C equations in the literature reflects the importance of LDL-C in clinical medicine and the need for greater accuracy in LDL-C estimation given new therapies and new guideline recommendations. In our study, most proposed alternatives to the Friedewald equation worsened accuracy of LDL-C. Clinical use of LDL-C equations without appropriate validation using ultracentrifugation could result in geographic disparities in cardiovascular care. Of 23 LDL-C equations evaluated in 5,051,467 patients, the Martin/Hopkins equation was the most accurate.

## Additional File

The additional file for this article can be found as follows:

10.5334/gh.1214.s1 Supplemental Tables and Figure. Supplemental Table 1 to 4 and Figure 1.
