# Supplementary material for: Accuracy of 23 Equations for Estimating LDL Cholesterol in a Clinical Laboratory Database of 5,051,467 Patients
Source: Glob Heart. 2023 Jun 19;18(1):36. doi: 10.5334/gh.1214 (PMC10289049; doi:10.5334/gh.1214)

Supplemental Table 1: LDL-C equations identified by systematic literature review

Lead author	Year	Formula	Country	Sample size (n)	Year of LDL-C measurement	LDL-C reference method
Friedewald	1972	TC – HDL-C – TG/5	United States	448	Unclear	β quantification
DeLong	1986	TC – HDL-C – (0.16 × TG)	North America	6,610	1972–1975	Ultracentrifugation
Rao	1988	[(4.7 × TC) – (4.364 × HDL-C) – TG]/4.487	Kuwait	196	Unclear	β quantification
Hattori	1998	(0.94 × TC) – (0.94 × HDL-C) – (0.19 × TG)	Japan	2,179	1992–1996	Ultracentrifugation
Puavilai	2004	TC – HDL-C – (TG/6)	Thailand	1,079	Not reported	Not reported
Anandaraja	2005	(0.9 × TC) – (0. 9 × [TG/5]) – 28	India	2,008	1998	Direct method (Beckman)
Teerakanchana	2007	(0.91 × TC) – (0.634 × HDL-C) – (0.111 × TG) – 6.755	Thailand	1,016	2004–2005	Direct method
Ahmadi	2008	TC/1.19 + TG/1.9 – HDL/1.1 – 38	Iran	230	2002–2003	Direct method (Technicon)
Saiedullah	2009	TC – TG/5–HDL-C + 15.3 (TG/TC) – 2.4	Bangledesh	Unclear	Not reported	Not reported
Chen	2010	(0.9 × TC) – (0.9 × HDL-C) – (0.1 × TG)	China	2,180	Not reported	Direct method (Roche)
Vujovic	2010	TC – HDL-C – (TG/6.85)	Serbia	2,053	2007–2008	Direct method (Kyowa Medex)
Cordova	2013	0.7516 × (TC – HDL-C)	Brazil	10,664	2000–2002	Direct method (Wako)

Martin/Hopkins	2013	TC − HDL-C − (TG / adjustable factor)	United States	1,350, 908	2009–2011	Vertical Auto Profile Ultracentrifuge (Atherotech)
Dansethakul	2015	0.9955 × TC – 0.9853 × HDL-C – 0.1998 × TG + 7.1449	Thailand	1,786	2008	Direct method (Roche)
Lee & Hu	2015	TC × 0.75–25	China	21,689	2010–2014	Direct method (Wako)
Rasouli	2017	0.75 × TC – 0.5 HDL – 0.1 × TG	Iran	310	Not reported	Direct method (Pars Azmon Inc)
Orejon, Ricra, Huallapa	2017	0.974 × TC – 0.160 × TG – 0.968 × HDL + 5.361	Peru	4,644	2015	Direct method (Siemens)
Ghasemi	2018	TC – HDL-C – TG/4	Iran	5,030	2012–2015	Direct method (Pars Azmon Inc)
Ephraim	2018	TC – HDL-C – TG/4	Ghana	1,518	2016–2017	Direct method
Bauer	2020	TC – HDL-C – TG/7.98	Germany	3,514	2014–2019	Direct method (Roche)
Molavi	2020	(0.97 × TC) – (0.93 × HDL-C) – (0.19 × TG)	Iran	3,844	2015	Not reported
Sampson	2020	TC/0.948 – HDL-C/0.971 – (TG/8.56 + [TG × Non-HDL-C]/2140 – TG^2/16100) – 9.44	United States	8,656	1976–1999	β quantification
Choi	2021	TC – 0.87 × HDL-C – 0.13 × TG	Korea	4,562	2017–2018	Direct method (Roche)

Supplemental Table 2: Percentage of patients correctly classified to LDL-C category for patients with triglyceride levels of 150–399 mg/dL

Equation	LDL-C category, mg/dL								
	**<40**	**40–54**	**55–69**	**70–99**	**100–129**	**130–159**	**160–189**	**≥190**	**Overall**
Martin/Hopkins	68.6	72.1	72.8	85.6	84.5	83.0	80.7	88.7	83.5
Sampson	39.3	42.8	51.9	78.3	82.3	83.3	81.8	89.0	79.3
Chen	63.5	64.7	67.3	82.3	82.0	81.4	80.3	92.8	81.3
Puavilai	31.5	37.4	49.9	80.3	85.2	84.2	78.5	80.5	79.4
DeLong	35.9	43.0	55.2	83.0	85.9	83.1	75.8	77.7	80.0
Friedewald	16.7	14.8	23.5	61.4	73.3	80.7	83.2	91.6	67.8

Supplemental Table 3: Percentage of patients correctly classified to LDL-C category for patients with triglyceride levels of 400–799 mg/dL

**Equation**	**LDL-C category, mg/dL**								
	**<40**	**40–54**	**55–69**	**70–99**	**100–129**	**130–159**	**160–189**	**≥190**	**Overall**
Extended M/H	59.0	45.7	43.6	65.2	60.8	58.4	56.8	71.4	60.3
Martin/Hopkins	41.3	41.5	42.1	65.4	61.0	57.4	54.3	65.9	59.2
Sampson	14.6	11.7	15.7	40.0	43.8	45.3	44.6	84.5	37.3
Chen	25.3	29.3	34.8	63.3	63.0	60.4	54.8	67.3	57.7
Puavilai	8.2	4.9	9.1	37.1	49.5	56.4	57.1	73.5	38.0
DeLong	9.4	6.6	12.1	42.1	53.8	58.9	57.4	70.7	42.2
Friedewald	4.4	1.1	2.5	15.6	26.5	36.6	43.5	83.0	18.2

Supplemental Table 4: Percentage of patients correctly classified to LDL-C category, stratified by subgroups

	Martin/Hopkins	Sampson	Chen	Puavilai	DeLong	Friedewald
Age category, y						
<18	91.4	87.6	87.5	83.8	83.1	83.9
18–59	89.6	85.9	84.0	83.8	83.0	83.1
≥60	89.5	86.8	84.8	84.7	83.8	83.2
Sex						
Women	90.4	87.1	84.0	85.2	84.3	84.7
Men	88.7	85.3	84.8	82.9	82.2	81.4
Fasting status						
Non-fasting	89.2	85.3	83.3	84.1	83.8	80.2
Fasting	90.4	87.5	84.8	85.0	83.9	85.3
eGFR, mL/min/1.73m^2						
<60	89.5	87.1	85.0	85.0	84.3	82.1
≥60	90.9	86.9	83.9	85.2	84.6	83.3
Diabetes						
Yes	88.6	84.8	83.7	84.0	84.1	76.8
No	89.6	86.3	84.4	84.1	83.3	83.4
Hypertension						
Yes	89.9	86.6	84.1	85.1	84.6	81.4
No	89.6	86.3	84.4	84.1	83.3	83.3
ASCVD						
Yes	90.1	87.2	86.1	84.5	84.2	81.0
No	89.6	86.3	84.3	84.1	83.3	83.2
High-sensitivity C-reactive protein, mg/L						
<2	91.4	87.2	85.2	84.7	83.8	85.0
≥2	89.9	86.8	83.3	85.4	84.8	82.1
TSH, uIU/mL						
<0.5	91.0	87.8	84.2	85.6	85.0	84.3
0.5–4.5	90.6	87.0	84.1	85.2	84.4	83.3
>4.5	89.7	86.9	83.3	85.3	84.8	82.5
Overall	89.6	86.3	84.4	84.1	83.3	83.2

Supplemental Figure 1: PRISMA chart for identification of relevant studies

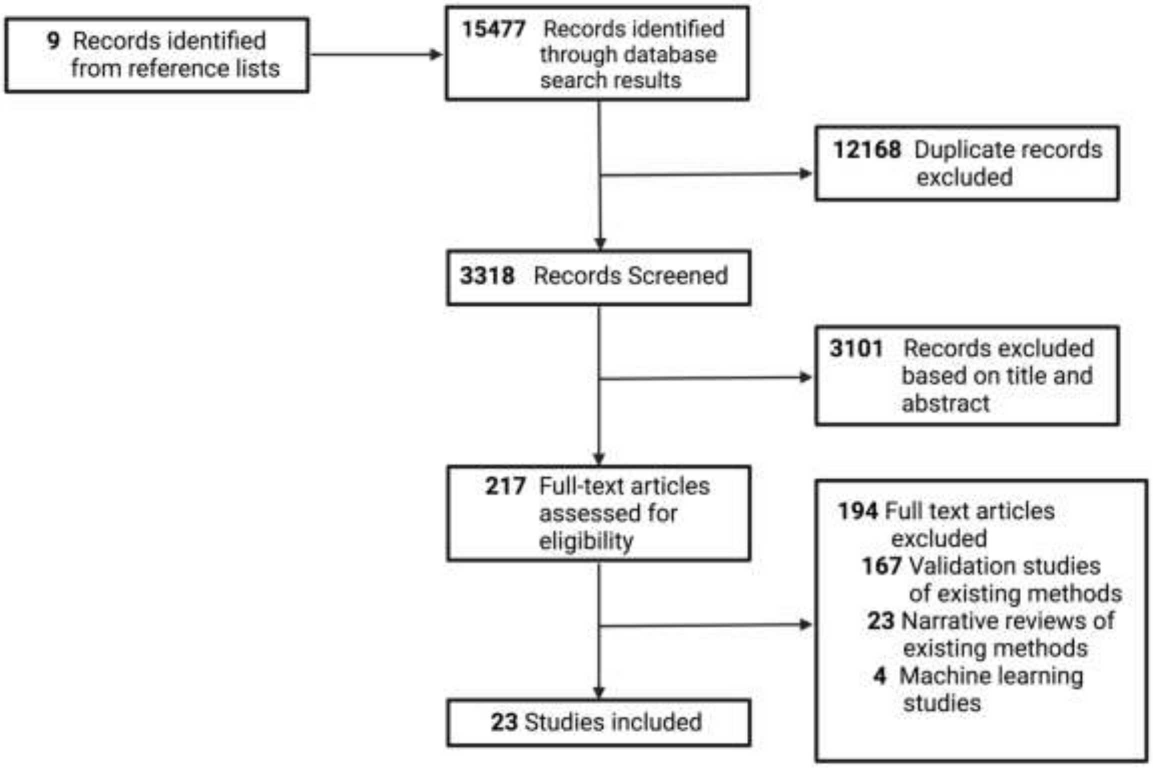

Supplement: Supplemental Tables and Figure. — Supplemental Table 1 to 4 and Figure 1. [file gh-18-1-1214-s1.pdf]
